# Preventive Antibiotic Prescribing Habits among Professionals Dedicated to Oral Implantology: An Observational Study

**DOI:** 10.3390/antibiotics10030301

**Published:** 2021-03-14

**Authors:** Angel Orión Salgado-Peralvo, Naresh Kewalramani, Juan Francisco Peña-Cardelles, María Victoria Mateos-Moreno, Loreto Monsalve-Guil, Álvaro Jiménez-Guerra, Iván Ortiz-García, Eugenio Velasco-Ortega

**Affiliations:** 1 Department of Stomatology, University of Seville, 41009 Seville, Spain; mmonsalve2@us.es (L.M.-G.); alopajanosas@hotmail.com (Á.J.-G.); ivanortizgarcia1000@hotmail.com (I.O.-G.); evelasco@us.es (E.V.-O.); 2 Science Committee for Antibiotic Research of Spanish Society of Implants (SEI–Sociedad Española de Implantes), 28020 Madrid, Spain; juanfranciscopenacardelles@gmail.com (J.F.P.-C.); mateosmoreno80@hotmail.com (M.V.M.-M.); 3 Department of Nursery and Stomatology, Rey Juan Carlos University, 28922 Madrid, Spain; k93.naresh@gmail.com; 4 Department of Basic Health Sciences, Rey Juan Carlos University, 28922 Madrid, Spain; 5 Department of Clinical Specialties, Faculty of Dentistry, Complutense University of Madrid, 28040 Madrid, Spain

**Keywords:** antibiotic prophylaxis, antibiotics, preventive antibiotics, antibiotic prescription behavior, prescribing trends, implant dentistry, oral implantology, dental implants

## Abstract

The prescription of preventive antibiotics (PA) in oral implantology is a controversial issue. The study aimed to determine the prescribing habits of PA in professionals dedicated to oral implantology in various treatments in healthy and at-risk patients. This is a cross-sectional observational study based on the STROBE (Strengthening the Reporting of Observational Studies in Epidemiology) guidelines. An electronic survey consisting of 4 blocks of questions was sent to members of the Spanish Society of Implants. The data were analyzed using descriptive analysis. A total of 303 participants (20.8%) responded to the questionnaire. One percent never prescribed PA, 55.4% prescribed them always, and 43.6% prescribed them sometimes. Ninety-six percent administered them preoperatively, while 92.4% administered them postoperatively. The most commonly used antibiotic is amoxicillin followed by amoxicillin with clavulanic acid (875/125 mg). Clindamycin is the most commonly administered antibiotic in patients with allergies. Professionals dedicated to oral implantology frequently prescribe PA in both healthy and at-risk patients, especially perioperatively. Immediate implant placement, sinus lifts, bone regeneration, and multiple implant placement are the treatments in which PA are most commonly prescribed, as well as in patients with heart valve prostheses or a history of bacterial endocarditis and immunodeficiency.

## 1. Introduction

Dental implants are the most predictable treatment option for total or partial replacement of missing teeth, however, around 0.7–3.8% of implants fail [1]. These failures can be “early” or “late” depending on whether they occur before or after functional loading, respectively [2]. Early failure occurs as a result of osseointegration failure due to local and/or systemic factors and accounts for 5% of all failures [3,4]. Since the beginning of oral implantology, the prescription of preventive antibiotics (PA) has been incorporated into implant placement protocols [5] due to the presence in the oral cavity of more than 500–700 bacterial species, in addition to other non-culturable microorganisms discovered by molecular biological techniques that can contribute to the development of postoperative infections [6,7].

Despite this, the routine prescription of preventive antibiotic therapy in healthy patients does not present a justified risk-benefit ratio [8,9,10]. The main reason for this is the growing worldwide development of bacterial resistance to virtually all known antibiotic families, which is making it increasingly difficult to treat infections due to the loss of efficacy of these drugs. The intrinsic complexity of antibiotic therapy decisions, poor microbiological information, and insufficient knowledge of infectious diseases can lead to poor selection or duration of antibiotic treatment and thus, to inappropriate use [11].

Antimicrobial resistance is, therefore, a major public health problem that causes around 33,000 deaths per year in the European Union [12] and the associated health care costs and lost productivity are estimated to be 1.5 billion euros per year [13]. It is a naturally occurring phenomenon, however, the inappropriate and indiscriminate use of antibiotics in humans, food-producing animals, and the environment is accelerating the process. Urgent changes in the way antibiotics are prescribed and used are needed because, even if new methods are developed, resistance will continue to pose a serious threat if current prescribing patterns are not modified [14].

Furthermore, antibiotics are used for longer periods in comparison with other drugs in oral surgery and implantology, such as anesthetics, analgesics, anti-inflammatory drugs, and anxiolytics, among others, which increases the risk of adverse reactions, such as allergies that may cause life-threatening effects [15,16]. In this regard, it is estimated that for every million patients administered a single dose of 3 g amoxicillin, fatal adverse reactions will occur in 0.1 and non-fatal in 4.7 [17]. These figures were considerably higher in other studies in which fatal reactions were reported in 0.9, severe in 400 and mild in 2400/million [18]. The most common alternative in penicillin-allergic patients is to prescribe clindamycin. In these cases, it is estimated that 11 fatal and 270 non-fatal reactions per million patients treated with 600 mg clindamycin will occur, most of them related to Clostridium difficile superinfection [17]. Other problems related to its use include direct toxicity including gastrointestinal problems (nausea, vomiting, diarrhea, and abdominal pain), hematological (neutropenia, thrombocytopenia, and hemolysis), nephrotoxicity (proteinuria or renal failure), neuropathies (nerve dysfunction or peripheral neuropathy), hepatobiliary disorders (jaundice or hepatitis), alterations in the usual bacterial flora of the mucous membranes (which can lead to yeast infections or pseudomembranous colitis), and drug interactions [19].

The present study aimed to analyze the guidelines for prescribing PA in various dental implant procedures in healthy patients and in those with risk factors by professionals dedicated to oral implantology, as well as to determine the factors that determine their decisions, to find out whether reasonable usage of these drugs is taking place and to raise awareness of the problems related to inappropriate prescription of these drugs.

## 2. Results

### 2.1. Participants

The survey was responded by a total number of 303 participants; thus, the response rate was 20.8%, which was considered an appropriate number.

### 2.2. Descriptive Data

The survey was answered by 219 men (72.3%) and 84 women (27.7%). The bulk of the participants ranged in age from 31 to 40 years (24.4%) and 41 to 50 years (23.4%). The majority of the people surveyed were dentists (75.6%) and, to a lesser extent, stomatologists (22.1%) and maxillofacial surgeons (2.3%). Most of them had studied a master’s degree related to oral implantology (61.1%) and had experience of up to 5 years in this type of treatment (28.7%) or more than 20 years of experience (30.7%). The professionals that participated had placed between 50 to 100 implants per year (57.4%) and did not practice exclusively in dental implant treatments (82.2%) (Table 1).

### 2.3. Main Results

Professionals dedicated to oral implantology prescribe PA to a large extent, with only 1.0% never prescribing them, while 55.4% always prescribe them and 43.6% “sometimes”.

In healthy patients, the most frequently used guideline is perioperative (36.3–52.2%). More complex treatments are the ones that most frequently demand this type of prescription, such as immediate implant insertion in the presence of infection of the tooth to be extracted (52.2%), bone regeneration and sinus lift with a lateral window (49.8%). The second most frequently used guideline is postoperative (11.6–33.0%), except for immediate implant placement in the presence of chronic infection of the tooth to be extracted, which is preoperative (17.5%). The majority of the participants did not prescribe antibiotics during the prosthetic phase of implants, i.e., in the second stage implant surgery (92.1%), during impressions and in implant prosthesis placement (95.1%) (Table 2).

Patients with a smoking habit, diabetes mellitus, immunodeficiency, hip prosthesis, heart valve prosthesis or at risk of infective endocarditis (IE), and/or psychiatric disorders were identified as risk factors. In these patients, the most commonly used guideline was also perioperative (25.4–67.0%), except in those with psychiatric disorders where the majority (43.2%) did not prescribe antibiotics. Patients with a history of IE and/or heart valve prosthesis (67.0%) and with immunodeficiency states (50.5%) are those in whom PA are most commonly prescribed (Table 3).

Preoperative antibiotics are prescribed by 96.0% of the professionals surveyed. Of these, the majority (39.5%) start treatment two days before, followed by one day before (35.1%) and only 25.4% prescribe them one hour before or immediately prior to surgery. The most commonly used antibiotic one or two days before surgery is amoxicillin (58.5%), specifically 750 mg (32.7%) TID (32.2%), followed by amoxicillin/clavulanic acid (40.1%) at a dose of 875/125 mg (34.1) TID (25.8%). Other antibiotics such as azithromycin or clindamycin are only prescribed by 1.4%. In prescribing one hour before or immediately prior to surgery, the most commonly prescribed antibiotic continues to be amoxicillin (87.9%), and the most commonly prescribed dose is 2 g (52.7%), followed by 1 g (27.0%).

Postoperative antibiotics are prescribed by 92.4% of participants. Of these, the majority use them for 7 (58.6%) or 5 days (31.8%). The most commonly prescribed is amoxicillin (55.7%) 750 mg (38.2%) TID (34.6%), followed by amoxicillin/clavulanic acid (41%) 875/125 mg (32.1%) TID (26.1%). Other antibiotics such as azithromycin (1.1%), clindamycin (1.8%) or erythromycin (0.4%) are prescribed by 3.2%. In penicillin-allergic patients, more than half of the surveyed professionals use clindamycin (58.4%), followed by azithromycin (22.1%) (Table 4).

When analyzing the factors that motivate PA prescribing habits, it is observed that those associated with scientific evidence, such as knowledge acquired in postgraduate courses (4.40 ± 0.86), during dental or medical studies (4.03 ± 1.06), reading scientific material (4.00 ± 1.13), or knowledge acquired in courses and congresses (3.95 ± 1.07) have a greater weight than those not associated to scientific evidence, such as the use of the antibiotic that the patient has at home (1.18 ± 0.55), recommendations from commercial firms (1.32 ± 0.64), the cost of the antibiotic (1.46 ± 0.92), recommendations from other colleagues (2.66 ± 1.17), or previous experience of the antibiotic in a similar procedure (3.72 ± 1.21) (Table 5).

## 3. Discussion

### 3.1. Key Results

The prescription of PA in dental implant surgeries is very common. Between 72.0–85.5% of clinicians in Finland, India, Sweden, the UK, and the USA routinely prescribe them pre-and/or post-operatively [19,20,21,22,23,24]. According to a study by Suda et al. [25], only 8.2% of PA prescriptions for dental exodontia, implant, or periodontal surgery are appropriate.

There are now recommendations for its prescription in placement of single implants [9,10,26,27,28,29,30] and bone regeneration procedures [14] in healthy patients. A recent network meta-analysis [31], which allows more than two interventions to be compared simultaneously (being the only better alternative a randomized clinical trial with several thousand participants, which would be quite complex) recommended the prescription of 2–3 g amoxicillin one hour before dental implant surgeries in ordinary situations in healthy patients. In the present study, only 10.2% prescribe them preoperatively in these cases.

Several systematic reviews and meta-analyses [9,10,26,27,28,29,30] have estimated the NNT (or “number needed to treat”), i.e., the number of individuals who must be treated with PA to prevent implant failure in one of them, from 24 [26] to 55 [28] and only one of every 143 will be prevented from a postoperative infection [28] therefore, making the prescription of PA a controversial issue at present. An expert committee concluded at the 4th Consensus Conference of the European Association for Osseointegration (EAO) [32] that preventive antibiotic therapy should not be systematically recommended in healthy patients, so the 23.4–15.4% of participants who do not prescribe them cannot be considered to be adopting the wrong approach. A high number of practitioners prescribe antibiotics inappropriately and/or overprescribe them either perioperatively (36.3%) or only postoperatively (30.0%).

On the other hand, the first systematic review that establishes a clear guideline on antibiotic prophylaxis in bone regeneration with one- or two-stage implant insertion has been recently published and it recommends the prescription of 2–3 g of amoxicillin one hour preoperatively [14]. Thus, only 13.2% of the professionals participating in the survey prescribed them adequately, while 77.2% administered them inadequately, either not using them at all (4.6%) or using them postoperatively (27.4%) or perioperatively (49.8%). On the other hand, only 25.4% of the professionals who prescribe PA preoperatively administer them 1 h before or immediately before the operation. In these cases, the most commonly prescribed antibiotic is amoxicillin (87.9%), and the most commonly administered dose is 2 g (52.7%). The 3 g dose was only administered by 4.1% of the professionals surveyed.

Concerning the rest of the implant procedures and risk conditions, there are currently no clear guidelines, so it is not possible to determine the suitability of the prescriptions carried out by the professionals surveyed, which explains the prolonged antibiotic treatment times reflected in the present study.

In the last 20 years, several studies have been published on antibiotic prescription patterns in oral implantology [18,19,20,24,33,34,35,36,37,38], mainly in the placement of single-tooth implants in healthy patients. The novelty of the present investigation is that it is the first study to investigate the patterns of PA administration in patients with at-risk conditions. Furthermore, only two articles have been published previously that inquired specifically about treatment guidelines for specific implant procedures [18,35] and the motivations behind these decisions [18,20].

With regard to the latter, as the age of the surveyed professionals increases, the knowledge acquired in postgraduate studies has less and less influence on their decision, and the cost of the antibiotic, although generally of little importance, has more and more weight. Basic university training was related to the age of the respondents, so that for dentists, the knowledge acquired during postgraduate training has a higher weight than for stomatologists and maxillofacial surgeons (*p* < 0.05), being the case opposite for the cost of the antibiotic (*p* < 0.05). For respondents who were trained in oral implantology through training courses, the knowledge acquired in postgraduate courses is significantly less important than for those who studied a master’s degree first (4.03 vs. 4.67; *p* < 0.05), followed by those who studied a master’s degree (4.45; *p* < 0.01) and those who completed any postgraduate course (4.41; *p* < 0.05). As the number of years of experience in these treatments increases and more implants are placed per year, the knowledge acquired during basic university studies, postgraduate studies and the recommendations of other colleagues gradually lose importance, probably due to the fact that more years have passed since these training courses and the experience has replaced the recommendations of other colleagues in the profession.

In the present study, the motivational factors described by AbuKaraky et al. [18] were taken as a reference, so that they can be compared and the results are similar since in both studies, the respondents gave greater importance to factors related to scientific evidence and experience. More specifically, in the survey of Jordanian dentists, reading scientific material is the most valued factor (86.6%), closely followed by knowledge acquired in graduate and postgraduate studies (86%), in courses and congresses (84.9%), and by previous experience with antibiotics (84.3%). The least valued factors were the influence of advertising (product samples, sales representatives, etc.) (16.3%), the availability of the antibiotic in the nearest pharmacy (24.4%), patient preferences (25%), the cost of the antibiotic (36%), and recommendations from other fellow professionals (43.0%). On the other hand, in a survey of dentists in the UK [20], the most important factor was the prevention of surgical site infection (84.4%). Alarmingly, only 30.3% of the respondents based their decisions on knowledge related to available scientific evidence, 16.5% on published guidelines and 47.7% on knowledge acquired at the postgraduate level. Slightly more than half of the respondents (51.4%) prescribe antibiotics to reduce bacteremia secondary to an infection, and recommendations from commercial firms are of importance for only 3.7%.

### 3.2. Limitations

As this is a survey-based study, it is not possible to establish with certainty the veracity of the answers provided by the participants. Furthermore, it is complex to determine which guidelines carried out by the participants are the most appropriate depending on the type of treatment considered since, so far, there is only scientific evidence of sufficient depth regarding the placement of implants in ordinary situations and simultaneously or not to bone augmentation procedures in healthy patients.

## 4. Materials and Methods

A cross-sectional observational study was carried out following STROBE (Strengthening the Reporting of Observational Studies in Epidemiology) guidelines [17]. Prior to the study, approval was obtained from the Ethics Committee of the Spanish Society of Implants (SEI—*Sociedad Española de Implantes*).

A questionnaire used by other studies [18,19] was modified in order to obtain more detailed information (File S1) on the patterns of prescribing PA in implant procedures in dentists dedicated to Oral Implantology. The questionnaire was sent via Google Drive and was open to respondents from April to July 2020, during which time two reminders were sent so that those who had not answered the questionnaire could do so.

The questionnaire is composed of 19 close-ended questions grouped into 4 blocks. The first block, composed of 7 questions, investigated general data concerning the surveyed professionals (demographic, academic, and professional data). The second block, with three multiple-choice questions, aimed to determine the frequency of prescribing according to different scenarios (implant procedures and patients with risk conditions). The third block, with three multiple-choice questions, studied the type of antibiotic and posology in healthy patients without allergies according to the regimen (pre-or postoperative), including a question on the antibiotic of choice in patients allergic to penicillin. The last block, consisting of a multiple-choice question, addressed the motivations for prescribing these drugs in implant treatments. All the questions were compulsory, as without answering one question it was not possible to move on to the next one.

The survey was sent to all members of the SEI who did not express their wish not to receive e-mails (*n* = 1.460) via the following link: https://docs.google.com/forms/d/1N-WABHszYrfKyvFLQwXDLVUHPi-ueLNmeQ8Z3gxPqLg/edit (accessed on 23 April 2020 to 30 July 2020). Completion of the survey implied the participant’s consent to the collection of this information. The final sample size comprised the professionals who decided to completely respond to the survey (*n* = 303). Each respondent could only answer one electronic survey once, and the options for each question, as well as the variables of the questionnaire, are shown in Table 1, Table 2, Table 3, Table 4 and Table 5.

There could not be any selection bias as the electronic survey was sent to all registered dentists in the SEI. Similarly, the authors employed two electronic surveys previously performed in the United States and in Jordan to avoid information bias.

The collected data were analyzed using IBM^®^ SPSS Statistics v.26 (IBM^®^ Corp., Armonk, NY, USA); 95% confidence intervals (CI) were used to assess the frequency of prescription for each antibiotic regimen. All descriptive variables of the subjects were determined as crossover variables (Table 1). All study variables were treated quantitatively. A normality test was previously applied, observing that no variable followed a normal distribution, so the Mann–Whitney U test was applied for the crossover for dichotomous variables and Kruskal–Wallis for variables with more than two categories. Factors determining the decision to prescribe antibiotics were treated in a qualitative manner. The chi-squared test was used.

## 5. Conclusions

Professionals dedicated to oral implantology frequently prescribe PA, especially perioperatively, however, in the absence of recommendations and/or sufficient evidence, it is not possible to establish the suitability of the prescribing habits described. The most commonly prescribed antibiotic is amoxicillin, while in those allergic to penicillin, it is clindamycin. The treatments that most frequently require the use of these drugs are the placement of immediate implants, especially if there is a chronic periapical infection, sinus lifts, bone regeneration, and multiple implant placement. In patients at risk, they are mainly used in patients with heart valve prostheses or a history of IE and immunodeficiencies, and to a lesser extent, in smokers and patients with mental disorders. In order to reduce the total dosage of antibiotics prescribed and thus the risk of emergence of antimicrobial resistance, it is necessary to establish clear recommendations for various implant procedures in both healthy and at-risk patients.

## Figures and Tables

**Table 1:** Demographic and professional characteristics of the study sample.

Variable	Specifications	N	%	95% CI
Gender	Male	219	72.3	67.8–76.8
	Female	84	27.7	23.2–32.2
Age (years)	<30	51	16.8	13.1–20.5
	31–40	74	24.4	20.1–28.7
	41–50	71	23.4	19.2–27.6
	51–60	57	18.8	14.9–22.7
	>60	50	16.5	12.8–20.2
University Basic Studies	Dentistry degree (Old plan)	170	56.1	15.5–23.5
	Dentistry degree (Bologna Plan)	59	19.5	51.1–61.1
	Stomatology	67	22.1	17.9–26.3
	Maxillofacial surgeon	7	2.3	0.8–3.8
Implant Education	Master´s Degree	185	61.1	56.2–66.0
	University Specialist Degree	69	22.8	18.6–27.0
	Postgraduate certificates (clinical stays, courses of commercial firms, etc.)	34	11.2	8.0–24.4
	Master´s Degree students	15	5.0	2.8–7.2
Experience with DIs (in years)	<5	87	28.7	24.2–33.2
	6–15	68	22.4	18.2–26.6
	16–20	55	18.2	14.3–22.1
	>20	93	30.7	26.1–35.3
Main Number of DIs Placed Per Year	<50	59	19.9	20.1–28.7
	50–100	170	57.4	22.0–30.8
	>100	67	22.6	44.2–54.2
Exclusive Clinical Practice in Dental Implant Treatments.	Yes	54	17.8	14.0–21.6
	No	249	82.2	78.4–86.0

CI, confidence interval; DIs, dental implants; N., sample size.

**Table 2:** Antibiotic prescription choices in different dental implant procedures in healthy patients.

Procedure	Antibiotic Choice									
	I Do Not Prescribe ATB		I Prescribe Only Pre-Op ATB		I Prescribe Only Post-Op ATB		I Prescribe Pre- & Post-Op ATB		I Do This Treatment	
	N (%)	95% CI	N (%)	95% CI	N (%)	95% CI	N (%)	95% CI	N (%)	95% CI
Single DI	71 (23.4)	19.2–27.6	31 (10.2)	7.2–13.2	91 (30.0)	25.4–34.6	110 (36.3)	31.5–41.1	303 (100.0)	100.0–100.0
Multiple DIs	33 (10.9)	7.8–14.0	34 (11.2)	8.0–14.4	93 (30.7)	26.1–35.3	143 (47.2)	42.2–52.2	303 (100.0)	100.0–100.0
Immediate DI placement in absence of active infection	43 (14.2)	10.9–18.1	32 (10.6)	7.6–14.0	97 (32.0)	27.9–37.5	125 (41.3)	37.1–47.1	297 (98.0)	96.6–99.4
Immediate DI placement in presence of active infection	6 (1.9)	0.7–4.1	53 (17.5)	16.4–25.6	35 (11.6)	10.0–17.8	158 (52.2)	57.3–68.1	252 (83.2)	79.5–86.9
Transcrestal sinus floor elevation	26 (8.6)	6.1–12.1	35 (11.6)	8.8–15.6	100 (33.0)	29.9–39.7	126 (41.5)	38.8–49.0	287 (94.7)	92.5–96.9
Lateral wall sinus floor elevation	13 (4.3)	2.4–6.8	40 (13.2)	10.6–18.0	76 (25.1)	22.4–31.8	151 (49.8)	48.6–59.2	280 (92.4)	89.7–95.1
Bone augmentation	14 (4.6)	2.7–7.1	40 (13.2)	10.3–17.5	83 (27.4)	24.1–33.5	151 (49.8)	47.2–57.6	288 (95.0)	92.8–97.2
Healing abutment placement	279 (92.1)	90.8–95.8	3 (1.0)	0.0–2.0	7 (2.3)	0.8–3.8	10 (3.3)	1.5–5.1	299 (98.7)	97.6–99.8
At time of impression making	288 (95.1)	94.8–98.4	2 (0.6)	0.0–1.5	2 (0.6)	0.0–1.5	6 (1.9)	0.6–3.4	298 (98.3)	97.0–99.6
At time of Crown placement	288 (95.1)	94.8–98.4	1 (0.3)	0.0–0.9	3 (1.0)	0.0–2.0	6 (1.9)	0.6–3.4	298 (98.3)	97.0–99.6

CI, confidence interval; DIs, dental implants; N., sample size; ATB, antibiotics; Pre-Op, preoperative; Post-Op, postoperative.

**Table 3:** Antibiotic prescription choices in patients with risk conditions.

Risk Condition	Antibiotic Choice									
	I Do Not Prescribe ATB		I Prescribe Only Pre-Op ATB		I Prescribe Only Post-Op ATB		I Prescribe Pre- & Post-Op ATB		I Treat Patients with this Condition	
	N (%)	95% CI	N (%)	95% CI	N (%)	95% CI	N (%)	95% CI	N (%)	95% CI
Smokers	107 (35.3)	31.0–40.8	21 (6.9)	4.4–9.6	61 (20.1)	16.4–24.6	109 (36.0)	31.7–41.5	298 (98.3)	97.0–99.6
Diabetes mellitus	49 (16.2)	12.6–20.0	37 (12.2)	9.0–15.6	66 (21.8)	17.8–26.2	148 (48.8)	44.3–54.3	300 (99.0)	98.0–100.0
Immunodeficiency disorders (antineoplastic treatment, lymphopenia, etc.)	15 (5.0)	3.2–8.4	51 (16.8)	15.4–24.2	38 (12.5)	10.9–18.7	153 (50.5)	54.1–64.9	257 (84.8)	81.2–88.4
Psychiatric disorders	131 (43.2)	42.7–53.3	18 (5.9)	3.9–9.3	47 (15.5)	13.2–21.2	77 (25.4)	23.4–33.0	273 (90.1)	87.1–93.1
IE and/or prosthetic hearth valve wearer	7 (2.3)	0.8–3.8	83 (27.4)	23.2–32.2	7 (2.3)	0.8–3.8	203 (67.0)	63.0–72.5	300 (99.0)	98.0–100.0
Hip prosthesis wearer	76 (25.1)	20.8–29.6	41 (13.5)	10.1–17.1	48 (15.8)	12.2–19.6	136 (45.0)	40.2–50.2	301 (99.3)	98.5–100.0

CI, confidence interval; DIs, dental implants; N., sample size; ATB, antibiotics; Pre-Op, preoperative; Post-Op, postoperative; IE, infective endocarditis.

**Table 4:** Summary response by participants.

Survey Question	Antibiotic Choice		
	Response	N (%)	95% CI
Do you routinely prescribe systemic antibiotic with DI placement?	Always	168 (55.4)	50.4–60.4
	Sometimes	132 (43.6)	38.6–48.6
	Never	3 (1.0)	0.0–2.0
**Preoperative prescribing habits**			
Do you prescribe antibiotics preoperatively prior to routine DI placement in healthy patients?	Yes	291 (96.0)	94.0–98.0
	No	12 (4.0)	2.0–6.0
If yes, when do you start prophylaxis prior to DI placement?	2 d prior	115 (39.5)	34.5–44.5
	1 d prior	102 (35.1)	30.2–40.0
	1 h prior or immediately prior	74 (25.4)	20.9–29.9
If 1- or 2-day(s) prior is selected	Amoxicillin 500 mg BID	1 (0.5)	0.0–1.4
	Amoxicillin 500 mg TID	30 (13.8)	9.6–18.0
	Amoxicillin 750 mg BID	1 (0.5)	0.0–1.4
	Amoxicillin 750 mg TID	70 (32.2)	26.6–38.0
	Amoxicillin 1.000 mg BID	18 (8.3)	4.9–11.7
	Amoxicillin 1.000 mg TID	7 (3.2)	1.0–5.4
	Amoxicillin/ clavulanic acid 500/125 mg BID	3 (1.4)	0.0–2.8
	Amoxicillin/ clavulanic acid 500/125 mg TID	10 (4.6)	2.0–7.2
	Amoxicillin/ clavulanic acid 875/125 mg BID	18 (8.3)	4.9–11.7
	Amoxicillin/ clavulanic acid 875/125 mg TID	56 (25.8)	20.4–31.2
	Azithromycin 500 mg QD	1 (0.5)	0.0–1.4
	Clindamycin 300 mg TID	2 (0.9)	0.0–2.1
If 1 h or immediately prior is selected	Amoxicillin 750 mg	3 (4.1)	0.0–8.5
	Amoxicillin 1.000 mg	20 (27.0)	17.1–36.9
	Amoxicillin 2.000 mg	39 (52.7)	41.6–63.8
	Amoxicillin 3.000 mg	3 (4.1)	0.0–8.5
	Amoxicillin/ clavulanic acid 500/125 mg	2 (2.7)	0.0–6.3
	Amoxicillin/ clavulanic acid 875/125 mg	7 (9.5)	3.0–16.0
**Postoperative prescribing habits**			
Do you prescribe antibiotics postoperatively after a routine DI placement?	Yes	280 (92.4)	89.7–95.1
	No	23 (7.6)	4.9–10.3
If yes, which antibiotic do you prescribe?	Amoxicillin 500 mg TID	49 (17.5)	13.5–21.5
	Amoxicillin 750 mg BID	10 (3.6)	1.6–5.6
	Amoxicillin 750 mg TID	97 (34.6)	29.6–39.6
	Amoxicillin/ clavulanic acid 500/125 mg BID	7 (2.5)	0.9–4.1
	Amoxicillin/ clavulanic acid 500/125 mg TID	18 (6.4)	3.8–9.0
	Amoxicillin/ clavulanic acid 875/125 mg BID	17 (6.1)	3.6–8.6
	Amoxicillin/ clavulanic acid 875/125 mg TID	73 (26.1)	21.5–30.7
	Azithromycin 500 mg QD	3 (1.1)	0.0–2.2
	Clindamycin 150 mg QID	1 (0.4)	0.0–1.1
	Clindamycin 300 mg TID	4 (1.4)	0.2–2.6
	Erythromycin (ethylsuccinate) 400 mg QID	1 (0.4)	0.0–1.1
How many days do you prescribe the antibiotic after the surgery (duration)?	1	2 (0.7)	0.0–1.6
	2	2 (0.7)	0.0–1.6
	3	18 (6.4)	3.8–9.0
	5	89 (31.8)	26.9–36.7
	7	164 (58.6)	53.4–63.8
	10	5 (1.8)	0.4–3.2
Which antibiotic do you prescribe in penicillin-allergic patients?	Clindamycin	177 (58.4)	53.5–63.3
	Azithromycin	67 (22.1)	17.9–26.3
	Erythromycin	57 (18.8)	14.9–22.7
	Clarithromycin	2 (0.7)	0.0–1.5

CI, confidence interval; DIs, dental implants; N., sample size; QD, once a day; BID, two times a day; TID, three times a day; QID, four times a day.

**Table 5:** Factors determining the prescription of antibiotics in oral implantology treatments ranked by preferences of the professionals surveyed.

Motivation	Mean (SD)	95% CI
Knowledge acquired during postgraduate training	4.40 ± 0.86	4.30–4.50
Knowledge acquired during basic university studies (dentistry/ stomatology)	4.06 ± 1.06	3.94–4.18
Scientific material reading	4.00 ± 1.13	3.87–4.13
Knowledge acquired in courses and/or congresses	3.95 ± 1.07	3.83–4.07
Previous experience with the antibiotic in a similar procedure	3.72 ± 1.21	3.58–3.86
Recommendations from other peers	2.66 ± 1.17	2.53–2.79
Patient preferences	1.68 ± 0.93	1.58–1.78
Cost of the antibiotic	1.46 ± 0.92	1.36–1.56
Recommendations from commercial companies	1.32 ± 0.64	1.25–1.39
Any antibiotic the patient may have at home	1.18 ± 0.55	1.12–1.24

SD, standard deviation; CI, confidence interval; 5, great importance; 4, quite important; 3, some importance; 2, little importance; 1, no importance.

## Data Availability

Data available in a publicly accessible repository.

## Supplementary Materials

The following are available online at https://www.mdpi.com/2079-6382/10/3/301/s1, File S1: Questionnaire.
