# Supplementary material for: Preventive Antibiotic Prescribing Habits among Professionals Dedicated to Oral Implantology: An Observational Study
Source: Antibiotics (Basel). 2021 Mar 14;10(3):301. doi: 10.3390/antibiotics10030301 (PMC7999193; doi:10.3390/antibiotics10030301)
Supplement: Supplementary file 1 [file antibiotics-10-00301-s001.pdf]

File S1. Questionnaire

Preventive antibiotic prescribing habits among professionals dedicated to Oral Implantology: An observational study - Questionnaire

<u>Block I:</u> **General variables related to the population surveyed** (demographic, academic, professional and experience data of the respondent).

1. **Gender:**
   - Male.
   - Female.
2. **Age (years):**
   - ≤ 30 years.
   - 31-40 years.
   - 41-50 years.
   - 51-60 years.
   - > 60 years.
3. **Education levels:**
   - Graduate in Dentistry.
   - Degree in Dentistry.
   - Stomatologist.
   - Maxillofacial surgeon
4. **Level of postgraduate training achieved:**
   - Non-accredited training courses (clinical placements, commercial firm courses, etc).
   - Postgraduate university courses related to Oral Implantology.
   - Student of any university master's degree related to Oral Implantology (Oral Surgery, Oral Implantology, Periodontics or combinations of these).
   - A completed master's degree course related to Oral Implantology.
5. **Years of experience placing implants:**
   - ≤ 5 years.
   - 6-15 years.
   - 16-20 years.
   - > 20 years.
6. **Approximate average number of implants placed per year:**
   - ≤ 50.
   - 51–100.
   - > 100.
7. **Are you dedicated exclusively to the placement of implants?**
   - Yes.
   - No.

<u>Block II:</u> **Frequency of prescription of different antibiotic regimens and regimens of choice according to different scenarios** (implant procedures and patients with risk or special conditions).

1. **In healthy patients, do you prescribe preventive antibiotics in dental implant surgeries?**
   - Always.
   - Sometimes.
   - Never.
2. **Please specify whether you prescribe preventive antibiotics in the following procedures in healthy patients:**
   - **2.1. Immediate implants:**
     - I do not normally prescribe antibiotics for this procedure.
     - I prescribe antibiotics preoperatively only.

- I prescribe antibiotics postoperatively only.
- I prescribe antibiotics preoperatively and postoperatively.
- I do not perform this type of treatment.

2.2. **Multiple implants:**
- I do not normally prescribe antibiotics for this procedure.
- I prescribe antibiotics preoperatively only.
- I prescribe antibiotics postoperatively only.
- I prescribe antibiotics preoperatively and postoperatively.
- I do not perform this type of treatment.

2.3. **Immediate implant placement without the presence of chronic infection in the site of the tooth to be extracted:**
- I do not normally prescribe antibiotics for this procedure.
- I prescribe antibiotics preoperatively only.
- I prescribe antibiotics postoperatively only.
- I prescribe antibiotics preoperatively and postoperatively.
- I do not perform this type of treatment.

2.4. **Immediate implant placement in the presence of chronic infection in the site of the tooth to be extracted:**
- I do not normally prescribe antibiotics for this procedure.
- I prescribe antibiotics preoperatively only.
- I prescribe antibiotics postoperatively only.
- I prescribe antibiotics preoperatively and postoperatively.
- I do not perform this type of treatment.

2.5. **Transcrestal (atraumatic) sinus lift:**
- I do not normally prescribe antibiotics for this procedure.
- I prescribe antibiotics preoperatively only.
- I prescribe antibiotics postoperatively only.
- I prescribe antibiotics preoperatively and postoperatively.
- I do not perform this type of treatment.

2.6. **Sinus lift with lateral window approach:**
- I do not normally prescribe antibiotics for this procedure.
- I prescribe antibiotics preoperatively only.
- I prescribe antibiotics postoperatively only.
- I prescribe antibiotics preoperatively and postoperatively.
- I do not perform this type of treatment.

2.7. **Bone augmentation procedures (bone regeneration):**
- I do not normally prescribe antibiotics for this procedure.
- I prescribe antibiotics preoperatively only.
- I prescribe antibiotics postoperatively only.
- I prescribe antibiotics preoperatively and postoperatively.
- I do not perform this type of treatment.

2.8. **Second stage implant surgery (uncovering implants prior to the beginning of the prosthetic phase):**
- I do not normally prescribe antibiotics for this procedure.
- I prescribe antibiotics preoperatively only.
- I prescribe antibiotics postoperatively only.
- I prescribe antibiotics preoperatively and postoperatively.
- I do not perform this type of treatment.

2.9. **Making impressions for implant-supported prosthesis:**
- I do not normally prescribe antibiotics for this procedure.
- I prescribe antibiotics preoperatively only.
- I prescribe antibiotics postoperatively only.
- I prescribe antibiotics preoperatively and postoperatively.
- I do not perform this type of treatment.

2.10. **Placement of the implant-supported prosthesis:**
- I do not normally prescribe antibiotics for this procedure.
- I prescribe antibiotics preoperatively only.
- I prescribe antibiotics postoperatively only.
- I prescribe antibiotics preoperatively and postoperatively.
- I do not perform these types of treatments.

3. **Mark if any of the following risk factors trigger the prescription of antibiotics:**

3.1. **Smokers:**
- I do not normally prescribe antibiotics for this situation.
- I prescribe antibiotics preoperatively only.
- I prescribe antibiotics postoperatively only.
- I prescribe antibiotics preoperatively and postoperatively.
- I do not treat this type of patient.

3.2. **Diabetes mellitus:**
- I do not normally prescribe antibiotics for this situation.
- I prescribe antibiotics preoperatively only.
- I prescribe antibiotics postoperatively only.
- I prescribe antibiotics preoperatively and postoperatively.
- I do not treat this type of patient.

3.3. **Immunodeficiency states (antineoplastic treatments, lymphopenia, convalescence or recent postoperative period, etc.):**
- I do not normally prescribe antibiotics for this situation.
- I prescribe antibiotics preoperatively only.
- I prescribe antibiotics postoperatively only.
- I prescribe antibiotics preoperatively and postoperatively.
- I do not treat this type of patient.

3.4. **Psychiatric disorders:**
- I do not normally prescribe antibiotics for this situation.
- I prescribe antibiotics preoperatively only.
- I prescribe antibiotics postoperatively only.
- I prescribe antibiotics preoperatively and postoperatively.
- I do not treat this type of patient.

3.5. **Patients with a history of bacterial endocarditis or prosthetic heart valves:**
- I do not normally prescribe antibiotics for this situation.
- I prescribe antibiotics preoperatively only.
- I prescribe antibiotics postoperatively only.
- I prescribe antibiotics preoperatively and postoperatively.
- I do not treat this type of patient.

3.6. **Patients with a hip prosthesis:**
- I do not normally prescribe antibiotics for this situation.
- I prescribe antibiotics preoperatively only.
- I prescribe antibiotics postoperatively only.
- I prescribe antibiotics preoperatively and postoperatively.
- I do not treat this type of patient.

<u>Bloque III:</u> Type of antibiotic of choice, dose and dosage of administration in healthy patients without allergies.

1. **Do you routinely prescribe antibiotics preoperatively and prior to implant procedures?**

1.1. **How many days before surgery do you start antibiotic prophylaxis?**
- 2 days before.
- 1 day before.
- 1 hour before.
- Immediately before.

- I never do preoperative prophylaxis.

1.2. If you have selected "1 or 2 days before", which antibiotic do you prescribe for a patient without allergies? (You can only select one type of antibiotic and one dose)

- I do not perform preoperative prophylaxis "one or two days before".
- *Amoxicillin:*
    - 500 mg, 2 times/day.
    - 500 mg, 3 times/day.
    - 750 mg, 2 times/day.
    - 750 mg, 3 times/day.
    - 1,000 mg, 2 times/day.
    - 1,000 mg, 3 times/day.
- *Amoxicillin/ clavulanic acid:*
    - 875/ 125 mg, 2 times/day.
    - 875/ 125 mg, 3 times/day.
    - 500/ 125 mg, 2 times/day.
    - 500/ 125 mg, 3 times/day.
    - 250/ 62.5 mg, 3 times/day.
- *Clindamycin:*
    - 150 mg, 4 times/day.
    - 300 mg, 4 times/day.
    - 300 mg, 3 times/day.
- *Erythromycin:*
    - 400 mg, 4 times/day (ethylsuccinate).
    - 800 mg, 2 times/day (ethylsuccinate).
    - 250 mg, 4 times/day (stearate).
    - 500 mg, 2 times/day (stearate)
- Azithromycin 500 mg, 1 time/day.

1.3. **If you have selected "one hour before" or "immediately before", which antibiotic do you prescribe in a patient without allergies?** (You can only select one type of antibiotic and one dose).

- I do not perform antibiotic prophylaxis "one hour before" or "immediately before" surgery.
- *Amoxicillin:*
    - 3,000 mg.
    - 2,000 mg.
    - 1,000 mg.
    - 750 mg.
    - 500 mg.
- *Amoxicillin/ clavulanic acid:*
    - 875/ 125 mg.
    - 500/ 125 mg.
    - 250/ 62.5 mg.
- *Clindamycin:*
    - 600 mg.
    - 300 mg.
    - 150 mg.
- *Erythromycin:*
    - 1,600 mg (ethylsuccinate).
    - 800 mg (ethylsuccinate).
    - 1.000 mg (stearate).
    - 500 mg (stearate).
- *Azithromycin:*
    - 1.000 mg.
    - 500 mg.

2. **Do you routinely prescribe antibiotics postoperatively for dental implant treatments in healthy patients??**
    - No.
    - Yes. If you have chosen this answer:

  2.1. **Which antibiotic do you prescribe in patients without allergies? (You can only select one type of antibiotic and one dose).**
    - I do not perform prophylaxis postoperatively.
    - *Amoxicillin:*
        - 250 mg, 4 times/day.
        - 500 mg, 3 times/day.
        - 750 mg, 2 times/day.
        - 750 mg, 3 times/day.
    - *Amoxicillin/ clavulanic acid:*
        - 500/ 125 mg, 2 times/day.
        - 500/125 mg, 3 times/day.
        - 875/ 125 mg, 2 times/day.
        - 875/ 125 mg, 3 times/day.
    - *Clindamycin:*
        - 150 mg, 4 times/day.
        - 300 mg, 3 times/day.
        - 300 mg, 4 times/day.
    - *Erythromycin:*
        - 400 mg, 4 times/day (ethylsuccinate).
        - 800 mg, 2 times/day (ethylsuccinate).
        - 250 mg, 4 times/day (stearate).
        - 500 mg, 2 times/day (stearate).
    - Azithromycin 500 mg, 1 time/day.

  2.2. **Duration of postoperative antibiotic treatment (in days):**
    - 1.
    - 2.
    - 3.
    - 5.
    - 7.
    - 10.
    - I do not perform postoperative prophylaxis.

3. **In patients allergic to beta-lactams (such as penicillin), which preventive antibiotic do you usually use?**
    - Clindamycin.
    - Azithromycin.
    - Clarithromycin.
    - Erythromycin.

Block IV: Factors affecting the decision to prescribe antibiotics.

1. **Which of the following factors affect the choice of antibiotic type and dosage of administration? (mark the degree of influence each factor has on your decision, with 1 being "none" and 5 being "very important")**
    - Patient preferences
    - Reading scientific material (articles, books, etc).
    - Knowledge acquired during dentistry/stomatology studies.
    - Knowledge acquired in postgraduate courses (specialisation courses, master’s degree or doctorate).
    - Knowledge acquired in courses and congresses.
    - Cost of antibiotics.
    - Recommendations from other fellow professionals.
    - Previous experience with the antibiotic in a similar procedure.
    - Recommendations from a commercial firm.

- I don't think it makes any difference. Any antibiotic that the patient keeps at home may be useful.
